# Intensity modulated radiation therapy following lumpectomy in early-stage breast cancer: Patterns of use and cost consequences among Medicare beneficiaries

**DOI:** 10.1371/journal.pone.0222904

**Published:** 2019-09-30

**Authors:** Lia M. Halasz, Shilpen A. Patel, Jean A. McDougall, Catherine Fedorenko, Qin Sun, Bernardo H. L. Goulart, Joshua A. Roth

**Affiliations:** 1 Department of Radiation Oncology, University of Washington, Seattle, Washington, United States of America; 2 Division of Public Health Sciences, Fred Hutchinson Cancer Research Center, Seattle, Washington, United States of America; 3 Department of Internal Medicine, University of New Mexico, Albuquerque, New Mexico, United States of America; 4 Hutchinson Institute for Cancer Outcomes Research, Fred Hutchinson Cancer Research Center, Seattle, Washington, United States of America; 5 Department of Medicine, Division of Medical Oncology, University of Washington, Seattle, Washington, United States of America; Centro per lo Studio e la Prevenzione Oncologica, ITALY

## Abstract

**Purpose:**

In 2013, the American Society for Radiation Oncology (ASTRO) issued a Choosing Wisely recommendation against the routine use of intensity modulated radiotherapy (IMRT) for whole breast irradiation. We evaluated IMRT use and subsequent impact on Medicare expenditure in the period immediately preceding this recommendation to provide a baseline measure of IMRT use and associated cost consequences.

**Methods and materials:**

SEER records for women ≥66 years with first primary diagnosis of Stage I/II breast cancer (2008–2011) were linked with Medicare claims (2007–2012). Eligibility criteria included lumpectomy within 6 months of diagnosis and radiotherapy within 6 months of lumpectomy. We evaluated IMRT versus conventional radiotherapy (cRT) use overall and by SEER registry (12 sites). We used generalized estimating equations logit models to explore adjusted odds ratios (OR) for associations between clinical, sociodemographic, and health services characteristics and IMRT use. Mean costs were calculated from Medicare allowable costs in the year after diagnosis.

**Results:**

Among 13,037 women, mean age was 74.4, 50.5% had left-sided breast cancer, and 19.8% received IMRT. IMRT use varied from 0% to 52% across SEER registries. In multivariable analysis, left-sided breast cancer (OR 1.75), living in a big metropolitan area (OR 2.39), living in a census tract with ≤$90,000 median income (OR 1.75), neutral or favorable local coverage determination (OR 3.86, 1.72, respectively), and free-standing treatment facility (OR 3.49) were associated with receipt of IMRT (p<0.001). Mean expenditure in the year after diagnosis was $8,499 greater (p<0.001) among women receiving IMRT versus cRT.

**Conclusion:**

We found highly variable use of IMRT and higher expenditure in the year after diagnosis among women treated with IMRT (vs. cRT) with early-stage breast cancer and Medicare insurance. Our findings suggest a considerable opportunity to reduce treatment variation and cost of care while improving alignment between practice and clinical guidelines.

## Introduction

The annual cost of cancer care in the United States is projected to reach approximately $175 billion by 2020, a 40% increase from 2010.[1] These escalating costs threaten the financial health of patients, health care providers and systems, payers, and the country. High costs also impact the ability for patients to receive recommended care and may increase their risk of death.[2] To slow the rising costs of cancer care, there is consensus across the cancer community that the value of cancer care should be considered.[3] This includes assessing the value of new technologies in regard to their marginal benefits, harms, and costs. These types of analyses are relatively common for pharmaceutical interventions, but are less frequently conducted in the case of radiation oncology interventions.

The American Society for Radiation Oncology (ASTRO), the largest radiation oncology professional society, has joined the growing ‘Choosing Wisely’ (CW) movement to highlight and curb the use of low-value radiation oncology technologies. CW is an effort initially spearheaded by the American Board of Internal Medicine that publicizes recommendations for avoiding low-value tests or procedures. In 2013, ASTRO issued a CW recommendation against the routine use of intensity modulated radiation therapy (IMRT) for whole breast radiation therapy.

Since first published in 1982, IMRT has become routinely available in radiation therapy centers across the United States and comprises about 20% of radiation treatments for all disease sites.[4] This radiation planning technique utilizes sophisticated dose optimization software and beam modulation to deliver highly conformal dose. Accordingly, Medicare reimbursement for IMRT procedures for breast cancer are greater than standard 3D conformal radiation therapy (cRT).

Proponents argue that IMRT allows for decreased radiation dose to the heart, which may lead to a lower rate of acute cardiac events.[5, 6] Long-term cardiac morbidity is an established late effect of radiation therapy for breast cancer which may have a significant impact on quality of life.[7–10] IMRT also allows for more homogenous dose distribution, avoiding areas of high radiation dose. Canadian and European trials have demonstrated decreased skin toxicity and better cosmetic outcomes with better dose homogeneity.[11–14] However, these trials often utilized simpler dose modulation technique than what is reimbursed as IMRT in the United States currently. A recently published small randomized trial of IMRT with deep inspiration breath hold (DIBH) compared to standard CRT for node-positive left-sided breast cancer showed better left ventricular ejection fraction one year after IMRT with DIBH.[15] Opponents argue that IMRT causes higher proportions of the heart and lungs receiving low doses of radiation therapy, which may increase risk of late effects including cardiac events, pulmonary disease, and secondary malignancy. However, there is a lack of evidence comparing late effects between alterative radiotherapy approaches.

Early reports on the increasing use of IMRT for breast cancer were published leading up to the ASTRO CW recommendation in 2013.[16–19] However, several Medicare carriers discontinued local coverage decisions (LCDs) that covered IMRT for breast cancer or changed LCDs to only allow IMRT in specific situations around 2008–2009.[20] Given these changes, the primary objectives of this study were to determine the baseline utilization of IMRT prior to the CW recommendation in 2013, to identify factors associated with CW recommendation adherence, and to estimate the impacts of IMRT use on breast cancer direct medical expenditure (vs. cRT).

## Methods and materials

### Data sources

All analyses used the Surveillance, Epidemiology, and End Results–Medicare (SEER-Medicare) database—a National Cancer Institute supported registry program that collects information on all cancer cases diagnosed in nine full states and nine additional regions. This analysis used data from the following registries that were available for the full analysis time horizon of 2008–2011: Connecticut, Detroit, California, Georgia, Hawaii, Iowa, Kentucky, Louisiana, New Jersey, New Mexico, Seattle, and Utah. SEER registries provide adjudicated sociodemographic and tumor characteristics variables, while the linked Medicare files provide longitudinal healthcare utilization data, including inpatient and outpatient hospital services, physician visits, laboratory, durable medical equipment, home health, and hospice services.[21] This analysis uses SEER information between 2008 and 2011 and Medicare claims from 2007 through 2012. The Institutional Review Board of Fred Hutchinson Cancer Research Center approved this study.

### Cohort selection

We included women diagnosed with breast cancer in a SEER region between July 1, 2007 and December 31, 2011, indicated by the International Classification of Diseases, 9th edition (ICD-9) codes for breast cancer (174.0–174.9). To study a cohort that received whole breast radiation therapy, we limited our cohort to Stage I and II, defined as T = 0–1, N = 0-1mi, M = 0 and T = 0–3, N = 0–1, M = 0, respectively using the American Joint Committee on Cancer TNM system Version 7.0.[22] We excluded patients under age 66 or without continuous enrollment in Medicare Part A (coverage of inpatient hospitalization, skilled nursing facility, and long-term care) and Part B (coverage of outpatient care) from 12 months prior to diagnosis to one year following diagnosis to ensure no prior breast cancer diagnosis and at least a year of follow-up for comorbidity and expenditure assessment, respectively. These women would not meet inclusion criteria for Medicare enrollment for a year prior to diagnosis, as most qualify for Medicare coverage at 65 years of age. Additionally, all included women received lumpectomy within 6 months of breast cancer diagnosis and then received radiation therapy within 6 months after lumpectomy. These restrictions were chosen to reflect typical clinical practice patterns and patients included in the CW recommendation.

### Clinical and demographic covariates

Additional clinical and demographic variables (Table 1) were derived from SEER records and used to conduct stratified analyses of IMRT use and as covariates in multivariable regression models. Sociodemographic factors, including race (white or non-white) and residence (Big Metropolitan versus small city/rural/other), were determined from SEER data. Socioeconomic quartiles were developed on the basis of median income or percent poverty in the census tract where the patient lived according to SEER data, using census tract data from the year 2010. Similarly, education quartiles were developed on the basis of percent college educated in the census tract where the patient lived. Comorbidities were identified by looking for diagnostic billing codes for specific health conditions during the year before the first diagnosis of breast cancer using the Deyo implementation of the Charlson score applied to inpatient and outpatient claims as described by Klabunde et al.[23–25]

**Table 1:** Clinical, demographic, and facility factors by receipt of intensity modulated radiation therapy following lumpectomy.

Variable	IMRT (n = 2580)	Conventional RT (n = 10457)	p-value
Age in Years at Index Date, Mean (SD)	74.5 (5.9)	74.4 (5.9)	0.44
	n (column %)		
Race			
White/Caucasian	2319 (89.9%)	9330 (89.2%)	0.30
Non-White/Caucasian	261 (10.1%)	1127 (10.8%)	
Residence			
Big Metro (>1 million people)	1764 (68.4%)	5585 (53.4%)	**<0.0001**
Other Setting (<1 million people)	816 (31.6%)	4872 (46.6%)	
Charlson Comorbidity Index			
0	916 (35.5%)	3528 (33.7%)	0.08
≥1	1664 (64.5%)	6929 (66.3%)	
Laterality			
Left breast	1552 (60.3%)	5034 (48.2%)	**<0.0001**
Right breast	1021 (39.7%)	5401 (51.8%)	
Tumor Stage			
Stage I	2019 (78.3%)	8242 (78.8%)	0.58
Stage II	561 (21.7%)	2215 (21.2%)	
Tumor Grade			
1	862 (33.4%)	3542 (33.9%)	0.33
2	1236 (47.9%)	4888 (46.7%)	
3	374 (14.5%)	1626 (15.6%)	
SEER Registry			
California	666 (25.8%)	3539 (33.8%)	**<0.0001**
Connecticut	126 (4.9%)	757 (7.2%)	
Detroit	392 (15.2%)	361 (3.5%)	
Georgia	391 (15.2%)	1088 (10.4%)	
Hawaii	0 (0%)	125 (1.2%)	
Iowa	24 (0.9%)	725 (6.9%)	
Kentucky	173 (6.7%)	548 (5.2%)	
Louisiana	181 (7.0%)	435 (4.2%)	
New Jersey	522 (20.2%)	1546 (14.8%)	
New Mexico	39 (1.5%)	211 (2.0%)	
Seattle	52 (2.0%)	842 (8.1%)	
Utah	14 (0.5%)	280 (2.7%)	
Facility type			
Hospital-based	1153 (44.7%)	7321 (70.0%)	**<0.0001**
Free-standing center	1427 (55.3%)	3136 (30.0%)	
LCD status			
Favorable	666 (25.8%)	3539 (33.8%)	**<0.0001**
Neutral	1809 (70.1%)	5585 (53.4%)	
Unfavorable	105 (4.1%)	1333 (12.8%)	
Median income in census track			
≤ $90,000	2280 (88.4%)	8681 (83.0%)	**<0.0001**
>$90,000	300 (11.6%)	1776 (17.0%)	
Poverty in census track			
≤ 25% living in poverty	2287 (88.6%)	9210 (88.1%)	0.48
>25% living in poverty	293 (11.4%)	1247 (11.9%)	
Education in census track			
≤ 50% college educated	2264 (87.8%)	8646 (82.7%)	**<0.0001**
> 50% college educated	316 (12.3%)	1811 (17.3%)	

Note: Bold p-values indicate statistically significant differences between cases and controls at α = 0.05

### Health services related variables

The SEER registries were grouped into three categories on the basis of the Carrier LCD governing Medicare coverage of procedures during the time period 2007–2011. The “favorable” coverage group had LCDs that explicitly allowed IMRT (California, Detroit and Georgia), even if some of the LCDs expired during the time period. The “unfavorable” coverage group only allowed IMRT for cardiac sparing (Hawaii and Seattle). The “neutral” group had no Carrier LCD that addressed IMRT for breast cancer (Connecticut, Iowa, Kentucky, Louisiana, New Jersey, New Mexico, and Utah). This classification approach was derived from a prior analysis of IMRT use in breast cancer.[17] Free-standing and hospital base facilities were identified by claims for delivery of radiation therapy in the Outpatient data file and Carrier Claims file, respectively.

### Outcome studied

The outcome variable of interest was IMRT receipt (yes vs. no), defined as having a claim for an IMRT specific code (77301;77418;0073T; or G6015-G6016). To identify women receiving any type of radiation therapy procedure following breast cancer diagnosis and receipt of lumpectomy, we queried Medicare inpatient and outpatient claims files for instances of procedure codes for radiation therapy (ICD-9 9220–9241, 77301, 77338, 77371–77373, 77385–77386,77401–77525;77761–77799;0073T;G0174;G0251;G0339-G0340;G6015-G06016;57155;58346;77750 and 0182T).

### Cost outcomes

Direct medical expenditures were calculated as Medicare allowable fees related to medical services that patients receive, including cancer treatment, hospitalization, physician visits, imaging, and laboratory testing. The Medicare allowable fees reflect both the component reimbursed by Medicare and any fees that patients must pay out-of-pocket. We calculated mean direct medical expenditure in the year after breast cancer diagnosis among women who received IMRT vs. cRT by summing all allowable Medicare fees (inpatient, outpatient, and carrier drug costs) in the 365 days after the index diagnosis date. This approach was intended to capture the cost consequences of radiotherapy modality, as well as associated resource use in the months following treatment. Expenditure outcomes were inflation-adjusted using the medical component of the consumer price index from the Bureau of Labor Statistics and reported in 2017 U.S. dollars.[26]

### Statistical analysis

We compared the characteristics of patients receiving IMRT and cRT using chi-square tests, Fisher exact test for categorical variables (where appropriate), and the Student t-test for continuous variables. We used a generalized estimating equations (GEE) logit model clustered on institution to evaluate differences in receipt of IMRT (vs. cRT) in unadjusted analyses and analyses adjusted for clinical, socio-demographic, and facility characteristics [27, 28]. GEE logit model estimates are similar to standard logistic regression models, but allow for dependence within clusters (e.g. institution where radiotherapy is provided). Our multivariable GEE logit models included age (continuous), race (white/other), residence in a ‘big metropolitan’ area (yes/no), breast cancer laterality (left/right), stage (1/2), grade (1/2/3), any comorbidities in the Charlson index (aside from cancer) (yes/no), census tract median household income >$90,000 per year (yes/no), census tract with >25% of residence having a high school degree or greater (yes/no), census tract with >25% living in poverty (yes/no), favorability of local coverage determination for IMRT coverage (favorable/neutral/unfavorable). We evaluated differences in mean direct medical expenditure for cases treated with IMRT vs cRT using the Mann-Whitney test.

## Results

### Cohort characteristics

**Fig 1:**
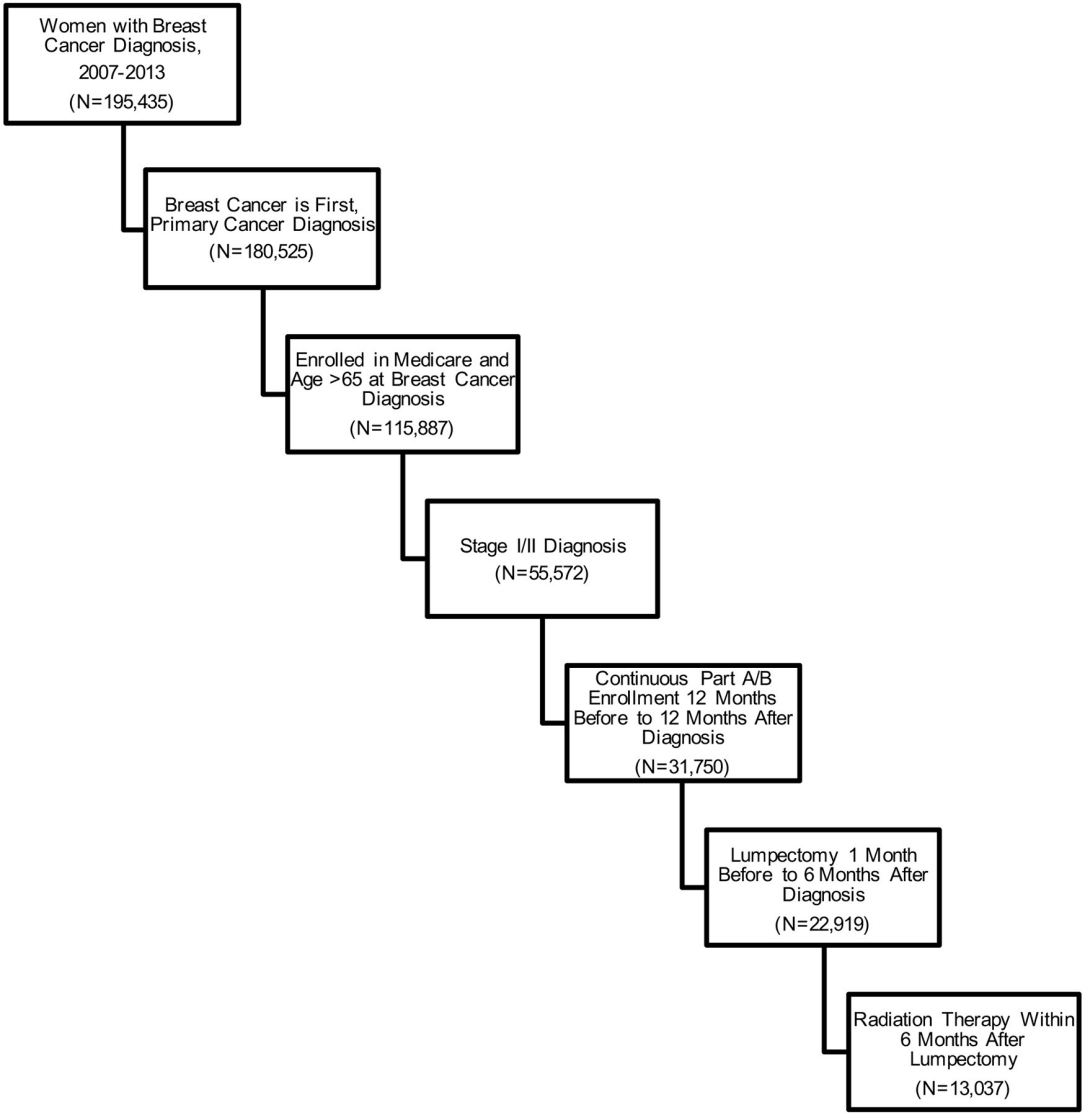
Study sample and inclusion/exclusion criteria.

Among a total of 195,435 breast cancer cases (any stage) identified between July 2007 and December 2011, 13,037 women met all inclusion criteria including receiving lumpectomy and radiation therapy for early stage breast cancer (Fig 1). Among this final cohort, the mean age was 74.4 years, 89% were white, and 50.5% had left-sided breast cancer (Table 1).

### Descriptive statistics for receipt of IMRT

**Fig 2:**
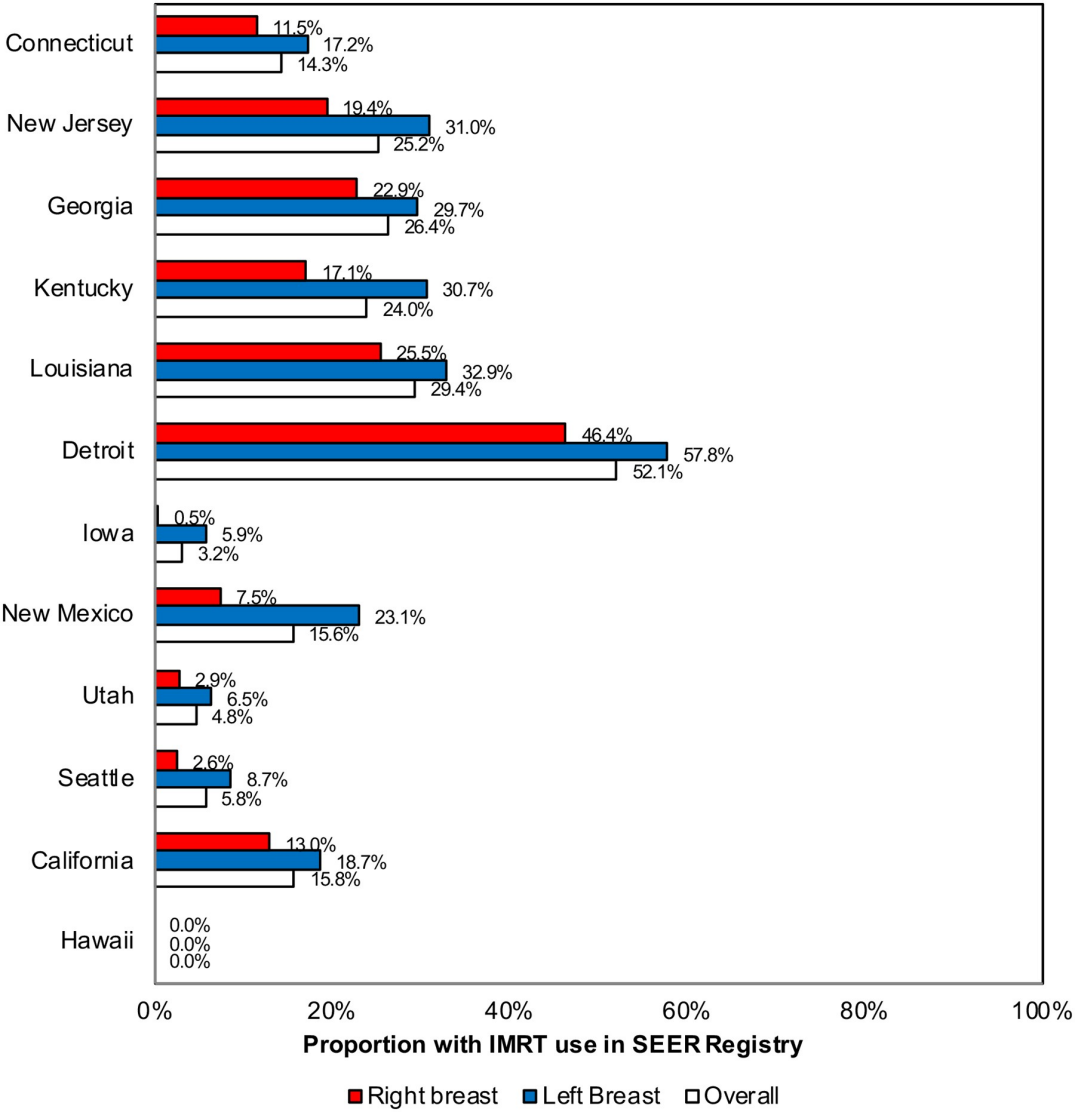
Proportion of early-stage breast cancer cases receiving intensity modulated radiation therapy following lumpectomy by Surveillance, Epidemiology, and End Results (SEER) registry in the SEER-medicare database.

Overall, 2581 women who received adjuvant radiation therapy (19.8%) received IMRT. Among women with left sided breast cancer, 1552 (23.6%) received IMRT compared to 1028 (15.9%) women with right sided breast cancer (P = <0.001). However, these proportions varied across regions—ranging from no women receiving IMRT in Hawaii to 52.1% receiving IMRT in Detroit (Fig 2).

### Factors associated with receipt of IMRT

In multivariable analysis (Table 2), the only clinical factor associated with IMRT use was left versus right sided breast cancer (OR 1.75; 95% confidence interval (CI) 1.59–1.92). Sociodemographic factors associated with IMRT included living in a Big Metropolitan Area (OR 2.39; 95%CI 2.16–2.64) or living in a census tract with ≤$90,000 median income (OR 0.57; 95% CI 0.49–0.66). However, health services factors were most strongly associated with IMRT use, including a neutral or favorable LCD (OR 3.86; 95%CI 3.12–4.76) and being treated at a free-standing facility (OR 3.49; 95%CI 3.17–3.83).

**Table 2:** Factors associated with IMRT use in multivariable logistic regression analysis. All variables listed were included in the multivariable model in addition to age at diagnosis (continuous), tumor stage (I or II), tumor grade (I, II, or III), race (white vs. other race), Charlson comorbidity score (0 vs. ≥1), residential setting (big metro vs. other setting), proportion living in poverty in census tract (≤25% vs. >25%), and proportion college educated in census tract (≤50% vs. >50%). Variables not shown did not have statistically significant associations with IMRT use.

Variable	Odds Ratio	95% Confidence Interval		P-Value
Left breast (vs. Right breast)	1.75	1.59	1.92	**<0.001**
Living in Big Metro Area (vs. Other)	2.39	2.16	2.64	**<0.001**
Living in census tract with <$90,000 median income (vs. >$90,000)	1.75	1.52	2.04	**<0.001**
Free standing facility (vs. Hospital)	3.49	3.17	3.83	**<0.001**
Neutral LCD (vs. unfavorable)	3.86	3.12	4.76	**<0.001**
Favorable LCD (vs. unfavorable)	1.72	1.38	2.15	**<0.001**

### Direct medical expenditures

In our analysis of direct medical expenditures in the year after breast cancer diagnosis among women treated with radiation therapy, we found that the direct mean expenditure in the year following breast cancer diagnosis varied significantly between registries (p<0.001). The average cost was $38,154 for women treated with IMRT and $29,655 for woman treated with cRT (Table 3). Accordingly, use of IMRT (vs. cRT) resulted in a statistically significant increase in direct medical expenditure of $8,499 (p = <0.001) in the year after breast cancer diagnosis.

**Table 3:** Mean one-year direct medical expenditure by SEER registry.

SEER Registry	N	% with IMRT	Mean Cost with IMRT	Mean Cost with cRT	Mean Cost Difference^
California	4,205	15.8%	$40,873	$31,889	**$8,984**
Connecticut	883	14.3%	$34,688	$30,580	**$4,107**
Detroit	753	52.1%	$36,755	$31,600	**$5,155**
Georgia	1,479	26.4%	$35,694	$28,626	**$7,068**
Hawaii	125	0.0%	*	$24,467	*
Iowa	749	3.2%	$33,905	$24,511	**$9,394**
Kentucky	721	24.0%	$36,204	$24,372	**$11,832**
Louisiana	616	29.4%	$33,409	$26,565	**$6,844**
New Jersey	2,068	25.2%	$41,790	$31,453	**$10,337**
New Mexico	250	15.6%	$30,751	$27,815	$2,936
Seattle	894	5.8%	$37,060	$28,387	**$8,674**
Utah	294	4.8%	$29,743	$26,459	$3,284
**All Registries**	**13,037**	**19.8%**	**$38,154**	**$29,655**	**$8,499**

^Bold values = statistically significant difference (p<0.01) in mean cost (IMRT vs. cRT)

*No patients received IMRT

## Discussion

We found that there was substantial IMRT use after lumpectomy (19.8%) in the years leading up to the ASTRO CW recommendation dissuading physicians from using IMRT for whole breast radiation therapy. Furthermore, the proportion of women receiving IMRT ranged from 0% to 52% across SEER registries. Our findings clearly demonstrate the need for the 2013 CW recommendation, as well how much room for improvement there is in reducing practice variation around IMRT after lumpectomy.

We also found that left-sided tumors were most strongly associated with IMRT use. This was not surprising as one often discussed advantage of IMRT is potential for decreased radiation dose to the heart in cases where the tumor is on the left side. However, there is a paucity of evidence demonstrating differential effects of IMRT vs. cRT on long-term cardiotoxicity, and so this use may not actually achieve the implied effect. Other factors strongly associated with IMRT use were Medicare LCD status and free-standing versus hospital-based billing—suggesting that financial incentives may encourage the selection of IMRT over cRT for breast cancer treatment following lumpectomy. In the case of Medicare LCD status, areas with “unfavorable” coverage policies have significantly less IMRT use, likely due to decreased likelihood of Medicare reimbursement for such services (vs. areas with “neutral” or “favorable” reimbursement policies). In the case of facility type, greater Medicare reimbursement rates for IMRT services provided at free-standing (vs. hospital-based) clinics may create an economic incentive for more use in those settings. Future hypothesis-driven studies should explore differential IMRT use by laterality, LCD status, and facility type to identify potential reimbursement and policy approaches to promote clinically appropriate use.

Our findings about IMRT use following lumpectomy are consistent with previous studies that evaluated the use of IMRT for adjuvant treatment of breast cancer, but the magnitude of difference in IMRT use in the current study was greater. Smith and colleagues estimated that IMRT use for women over age 65 increased from 0.9% in 2000 to 11.2% in 2005, and this use varied between <0.7% and 23.1% among SEER regions [17]. Roberts and colleagues also used the SEER-Medicare database to estimate a 12.6% rate of IMRT use in 2007.[18] Recently, Wang and colleagues published an analysis of the National Cancer Data Base (NCDB), reporting an increase in IMRT use from 5.3% in 2004 to 11.6% in 2009, with a decline to 10.7% in 2011.[16] However, NCDB represents a substantially different patient population than our population-based SEER-Medicare analysis. Specifically, NCDB is a data set based on facilities accredited by the American College of Surgeons and includes patients covered by many insurance types.

Lastly, we found that IMRT use was associated with significantly increased Medicare expenditure in the year after breast cancer diagnosis. Overall, mean direct medical expenditure for all patients in the year after diagnosis was $31,337 (95% CI = $31,034-$31,641), and women treated with IMRT had mean expenditure that was 28.7% higher ($38,154, 95% CI = $37,432-$38,876) vs. cRT ($29,655, 95% CI = $29,328-$29,981). Registries with the most IMRT usage had the highest expenditures, while registries with little or no IMRT use had lower mean expenditures. When weighted to be nationally representative by age and region, we estimate that this degree of IMRT overuse amounts to an additional $116 million in annual Medicare expenditures. Assuming no clinically meaningful benefits of IMRT over cRT, U.S. healthcare systems could save substantial resources if providers adhere to the CW recommendation addressed by this study. Alternatively, if IMRT could plausibly confer clinical benefits, a rationale would exist for a pragmatic randomized trial comparing IMRT and cRT, with patient- and societal-centered outcomes, including symptoms, cardiac morbidity, and costs.

Smith and colleagues had estimated that the mean cost of radiation with IMRT was $15,230 from 2001–2005 (approximately $24,361 inflation adjusted to 2017 USD), while Roberts and colleagues estimated the mean cost of radiation with IMRT was $12,375 from 1998–2007 (approximately $20,586 inflation adjusted to 2017 USD).[17, 18] Our estimate of mean direct medical expenditure is much larger given it includes allowable costs related to medical services that patients receive, including cancer treatment, hospitalization, physician visits, imaging, and laboratory testing in the year after breast cancer diagnosis. Our approach to assessing the cost-consequences of alternative radiation therapy strategies has the advantage of capturing downstream impacts on healthcare resource utilization, thus providing a more comprehensive view of the economic consequences of IMRT use.

The observational study design and use of administrative data on Medicare enrollees presents several important limitations that should be considered in the interpretation and generalizability of these results. The outcomes studied were based on billing claims as a surrogate for clinical data, which may have led to misclassification. Additionally, we could not evaluate the effect of IMRT on survival, recurrence, or long-term toxicity due to the short follow up time and rare nature of these outcomes. Lastly, our sociodemographic descriptions were based on census tract rather than patient-level data, which may have led to ecologic fallacy and could explain the curious result that patients in rich census tracts (>$90,000 median income) were less likely to receive IMRT. Alternatively, this finding may describe a disparate overuse in less affluent communities which has been described for other technological advancements prone to overuse.[22]

Future studies will need to investigate whether high levels of IMRT persist despite lack of evidence of its clinical impact, especially after cost containment strategies such as CW were implemented. Interestingly, during the study period, multiple LCDs were ended in 2008 and 2009, leaving the regions with no policies regarding the coverage of IMRT for breast cancer. However, the trend of use following this change was not consistent between regions. The SEER-Medicare files were recently updated to include patients diagnosed through 2015, and so we plan to undertake a similar analysis looking at the change in IMRT ffrom before the 2013 ASTRO CW recommendation versus after the recommendation. These findings will further elucidate patterns of IMRT use over time in low-risk early-stage breast cancer, and will be able to inform stakeholders whether the high levels of IMRT use seen in the 2007–2011 period persisted after ASTRO CW recommendation.

## Conclusions

We found that a substantial proportion (19.8%) of women with early-stage breast cancer received IMRT after lumpectomy in the years leading up to an ASTRO CW recommendation against this practice. This led to an additional $8,499 in mean Medicare expenditure among patients receiving IMRT (vs. cRT) in the first year of breast cancer diagnosis, and this additional expenditure is not expected to yield any major clinical benefit. Future studies should ascertain the impact of the CW recommendation on IMRT use and evaluate interventions to improve adherence to clinical guidelines.
